# The heme-binding protein PhuS transcriptionally regulates the *Pseudomonas aeruginosa* tandem sRNA *prrF1,F2* locus

**DOI:** 10.1016/j.jbc.2021.100275

**Published:** 2021-01-09

**Authors:** Tyree Wilson, Susana Mouriño, Angela Wilks

**Affiliations:** Department of Pharmaceutical Sciences, School of Pharmacy, University of Maryland, Baltimore, Maryland, USA

**Keywords:** Heme, iron-metabolism, DNA-binding, bacterial pathogenesis, sRNA, 5′-FAM, 5′-fluoroscein amidite, Fur, ferric uptake regulator, BSA, bovine serum albumin, BV, biliverdin, ChIP, chromatin immunoprecipitation, ECF, extracytoplasmic function, FA, fluorescence anisotropy, gDNA, genomic DNA, GST, glutathione-*S*-transferase, Has, heme assimilation system, Ni–NTA, nickel–nitrilotriacetic acid, Phu, *Pseudomonas* heme uptake, PQS, *Pseudomonas* quinolone signal, qPCR, quantitative PCR

## Abstract

*Pseudomonas aeruginosa* is an opportunistic pathogen requiring iron for its survival and virulence. *P. aeruginosa* can acquire iron from heme *via* the nonredundant heme assimilation system and *Pseudomonas* heme uptake (Phu) systems. Heme transported by either the heme assimilation system or Phu system is sequestered by the cytoplasmic protein PhuS. Furthermore, PhuS has been shown to specifically transfer heme to the iron-regulated heme oxygenase HemO. As the PhuS homolog ShuS from *Shigella dysenteriae* was observed to bind DNA as a function of its heme status, we sought to further determine if PhuS, in addition to its role in regulating heme flux through HemO, functions as a DNA-binding protein. Herein, through a combination of chromatin immunoprecipitation–PCR, EMSA, and fluorescence anisotropy, we show that apo-PhuS but not holo-PhuS binds upstream of the tandem iron-responsive sRNAs *prrF1,F2*. Previous studies have shown the PrrF sRNAs are required for sparing iron for essential proteins during iron starvation. Furthermore, under certain conditions, a heme-dependent read through of the *prrF1* terminator yields the longer PrrH transcript. Quantitative PCR analysis of *P. aeruginosa* WT and Δ*phuS* strains shows that loss of PhuS abrogates the heme-dependent regulation of PrrF and PrrH levels. Taken together, our data show that PhuS, in addition to its role in extracellular heme metabolism, also functions as a transcriptional regulator by modulating PrrF and PrrH levels in response to heme. This dual function of PhuS is central to integrating extracellular heme utilization into the PrrF/PrrH sRNA regulatory network that is critical for *P. aeruginosa* adaptation and virulence within the host.

Bacterial pathogens must acquire iron from their host for survival and virulence where because of its reactivity, it is tightly regulated and sequestered in iron-binding proteins, such as transferrin and ferritin, or in heme and iron–sulfur cluster–containing proteins. Iron is further limited within the host during infection by the hosts' innate immune response that includes the secretion of high-affinity iron-binding proteins, such as lipocalin 2, and hepcidin-dependent downregulation of plasma iron levels (1). To circumvent this nutritional immunity, invading pathogens possess several acquisition strategies to acquire iron, and many encode systems for the utilization of heme (2, 3, 4, 5). The gram-negative opportunistic pathogen *Pseudomonas aeruginosa* encodes two heme uptake systems; the heme assimilation system (Has) and the *Pseudomonas* heme uptake (Phu) system (6). The Has and Phu systems were shown to have nonredundant roles in heme sensing and transport, respectively (7). The Has system encodes an extracytoplasmic function (ECF) σ/anti-σ factor system, HasIS (6). ECF σ factors are a subfamily of alternative σ_70_ factors that allow for transcriptional amplification of genes involved in extracellular stress-response functions (8, 9). The secreted hemophore HasAp on interaction with the outer membrane receptor HasR triggers activation of the ECF σ/anti-σ factor system HasIS. However, heme transported by either the HasR or the PhuR outer membrane receptors is translocated to the cytoplasm by the *phu*-encoded ABC transporter PhuUV and its cognate periplasmic heme-binding protein PhuT. Previous studies have shown that the cytoplasmic heme-binding protein PhuS regulates the flux of heme into the cell through a specific interaction with the iron-regulated heme oxygenase, HemO (10, 11). HemO oxidatively cleaves heme to release iron, CO, and biliverdin (BV) IXβ and IXδ (12). Interestingly, the HemO metabolite BVIXβ and/or BVIXδ is a post-transcriptional regulator of HasAp protein levels (13). Thus, the PhuS–HemO couple regulates both the flux of heme into the cell and the extracellular heme signal through the heme metabolites, BVIXβ and IXδ.

The complexity of *P. aeruginosa* iron and heme homeostasis is further exemplified by the tandem arrangement of the PrrF1 and PrrF2 sRNAs found directly downstream of the *phu* operon (Fig. 1*A*) (14). The PrrF sRNAs are highly homologous to one another and contribute to iron homeostasis by causing mRNA degradation of nonessential iron-containing proteins (14, 15, 16). The PrrF sRNAs play a role in numerous other processes, including twitching motility, quorum sensing molecule production, and biofilm formation. Furthermore, this tandem arrangement allows for the expression of an overlapping noncoding RNA, PrrH, whose expression is heme dependent (17). The duplication of the *prrF* genes and the presence of *phuS* are genetically linked and found in pathogenic *P. aeruginosa* but not in other Pseudomonads (Fig. 1*A*) (17). Interestingly, PrrH is detected in infected murine lungs as well as sputum from patients with cystic fibrosis suggesting a role for this sRNA during infection (18). In addition, deletion of *phuS* or the *prrF1,F2* locus gave similar iron dysregulation transcriptomic profiles (15, 19). In addition, previous studies have shown that the *Shigella dysenteriae* ShuS, a homolog of PhuS, has DNA-binding properties (20). Based on these previous studies, we hypothesized that PhuS may also possess DNA-binding properties providing a functional link between PhuS and the *prrF1,F2* locus. To test this hypothesis, we performed a series of *in vivo* and *in vitro* experiments to determine the functional link between PhuS and *prrF* locus. Herein, through chromatin immunoprecipitation–PCR (ChIP–PCR), EMSA, and fluorescence anisotropy (FA), we show that apo-PhuS binds with high affinity to the promoter of *prrF1* but not that of *prrF2*. Furthermore, comparison of the relative expression of PrrF and PrrH in the PAO1 WT and Δ*phuS* allelic strains by quantitative PCR (qPCR) shows a loss in the heme-dependent regulation of PrrH in the absence of PhuS. We propose that PhuS has a dual function integrating extracellular heme metabolism into the iron-homeostasis networks through transcriptional modulation of the PrrF/PrrH sRNAs.

**Figure 1:**
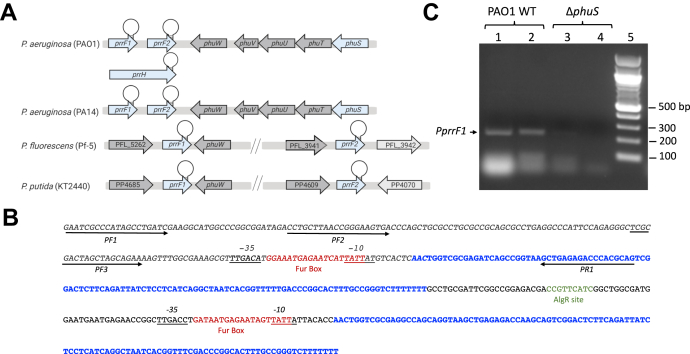
**Chromatin immunoprecipitation (ChIP)–PCR analysis of PhuS binding to the *prrF1,F2* promoter.***A*, genetic organization of the *prrF1,F2* locus in pathogenic and nonpathogenic *Pseudomonas* strains. *B*, sequence of the *prrF1,F2* locus. PrrF1 and PrrF2 are shown in *blue*. The ferric uptake regulator boxes upstream of *prrF1* and *prrF2* are shown in *red*, the AlgR site in *green*, and the -35 and -10 sites are *underlined*. Primers used in ChiP–PCR pull downs are indicated by the *black arrows*. The *italicized* sequence represents the 225 bp P*prrF1* fragment obtained following ChIP–PCR and DNAse I treatment. *C*, PCR fragments (225 bp; utilizing primers PF1 and PR1) isolated from PAO1 WT cells or Δ*phuS* control strain following crosslinking and pull down with anti-PhuS. Lane 1, PAO1 WT in iron-deplete media; lane 2, PAO1 WT supplemented with 1 μM heme; lane 3, Δ*phuS* iron-deplete media; lane 4, Δ*phuS* supplemented with 1 μM heme; and lane 5, DNA markers as shown. Bands were visualized on 1% agarose with ethidium bromide staining.

## Results

### Isolation of a PhuS–prrF1 complex by ChIP–PCR

The potential for PhuS to bind to the *prrF1* promoter was analyzed by subjecting *P. aeruginosa* (PAO1) WT or the Δ*phuS* deletion strain to ChIP followed by PCR amplification, employing primers specific for the *prrF1* promoter (Table S1). Following DNAse digestion, reversal of the formaldehyde crosslinking, and sequencing, we determined a ∼230 bp fragment upstream of the *prrF1/prrH* promoter that includes part of the ferric uptake regulator (Fur) box sequence (Fig. 1, *B* and *C*). We repeated the pull downs with purified genomic DNA (gDNA; 100–500 bp sheared fragments) and addition of purified His-tagged PhuS (PhuS-His_6_) to PAO1 WT. Nickel–nitrilotriacetic acid (Ni–NTA) pull down, DNAse treatment, and PCR amplification with the primer pairs shown in Figure 1*B* identified bands of ∼230, 180, and 120 bp, which following sequencing confirmed binding to the *prrF1* promoter (Fig. S1). Because of the high sequence identity (>95%) between PrrF1 and PrrF2, we were unable to design primers to specifically probe PhuS binding to the *prrF2* promoter. PhuS binding to the *prrF2* promoter was analyzed by FA (see the next section).

### Apo-PhuS and Fur have overlapping binding sites within the prrF1 promoter

**Figure 2:**
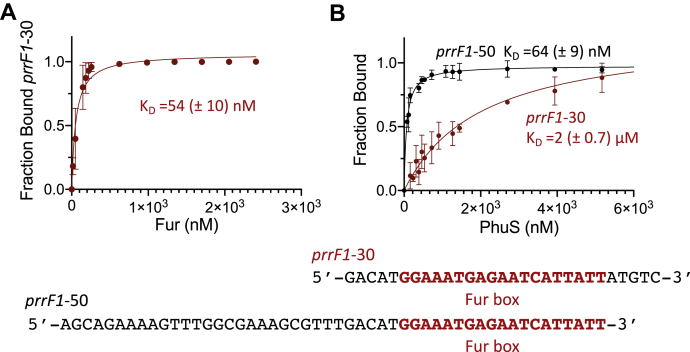
**Fluorescence anisotropy of ferric uptake regulator (Fur) and PhuS binding.***A*, Mn–Fur binding to the 5′-FAM–labeled *prrF1*-30. *B*, apo-PhuS binding to the 5′-FAM–labeled *prrF1*-50 and *prrF1*-30 color coded as shown. Experiments were performed in triplicate as described in Experimental procedures section. The data were fit by converting the anisotropy, *r*, to fraction bound and plotted against protein concentration using a one-site binding model. The error is shown as the SEM. 5′-FAM, 5′-fluoroscein amidite.

Interestingly, the DNA fragment isolated by ChIP–PCR included the *prrF1* Fur box (Fig. 1*B*). Utilizing FA, we analyzed Fur and PhuS binding to a 5′-fluoroscein amidite (5′-FAM)–labeled 30 bp oligonucleotide encoding the Fur box alone (*prrF1*-30) (Fig. 2*A* and Table S2). The change in anisotropy on addition of Fur when fit to a one-to-one binding site model gave a binding constant (*K*_*D*_) of 50 ± 10 nM (Fig. 2*A*). In contrast, addition of apo-PhuS to *prrF1*-30 showed a much smaller change in anisotropy with a *K*_*D*_ >2 μM (Fig. 2*B*). However, titration of a 5′-FAM–labeled oligonucleotide that includes sequence upstream of the Fur box (*prrF1*-50) significantly enhanced PhuS binding (Fig. 2*B* and Table S2). The change in anisotropy when fit to a one-to-one binding site model gave a *K*_*D*_ of 64 ± 10 nM (Fig. 2*B*). In contrast, a 5′-FAM–labeled oligonucleotide encompassing the upstream sequence but lacking the Fur box showed no change in anisotropy (Fig. S2*A* and Table S2). Therefore, the optimal binding of PhuS to the *prrF1* promoter requires sequence upstream of and including the Fur box. To confirm PhuS specificity for the *prrF1* promoter over that of *prrF2*, we performed FA on 5′-FAM–labeled oligonucleotides designed within the *prrF1,prrF2* intergenic region (Table S2). Incremental addition of apo-PhuS to 5′-FAM–labeled oligonucleotides *prrF*2-50 (Fur) including the Fur box or the upstream *prrF*2-50 (AlgR), which includes the AlgR site (Fig. 1*B* and Table S2), showed no change in anisotropy (Fig. S2*A*), confirming PhuS specificity for the *prrF1* promoter.

### Apo-PhuS but not holo-PhuS binds to the prrF1 promoter

**Figure 3:**
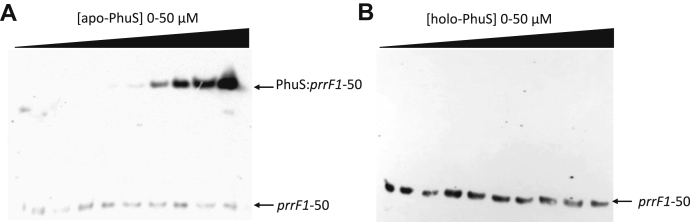
**EMSA of apo- and holo-PhuS binding to *prrF1*-50**. *A*, apo-PhuS binding to 5′-biotin–labeled *prrF1*-50. *B*, holo-PhuS binding to 5′-biotin–labeled *prrF1*-50. Experiments were performed as described in Experimental procedures section. All reactions contained a fixed concentration (30 pM) of labeled *prrF1*-50, and the following incubation was run on 8% acrylamide gels and transferred to a nylon membrane and visualized by chemiluminescence.

Following characterization of the PhuS-binding region, we next sought to determine if heme and DNA binding were mutually exclusive. In contrast to apo-PhuS, the addition of holo-PhuS to the 5′-FAM–labeled *prrF1*-50 oligonucleotide showed no change in anisotropy (Fig. S2*B*). The FA analysis was confirmed with EMSAs of apo-PhuS and holo-PhuS binding to a 5′-biotinylated *prrF1*-50 oligonucleotide. Addition of increasing concentrations of apo-PhuS gave a lower mobility complex consistent with apo-PhuS binding to *prrF1*-50 (Fig. 3*A*). In contrast, addition of holo-PhuS showed no shift in biotinylated *prrF1*-50 (Fig. 3*B*). Taken together, the data are consistent with heme and DNA binding being mutually exclusive functions of PhuS.

### HemO modulation of the holo-PhuS to apo-PhuS equilibrium drives DNA binding

**Figure 4:**
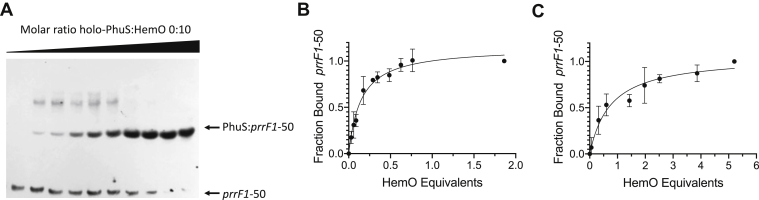
**Holo-PhuS titration with apo-HemO drives DNA binding**. *A*, EMSA of holo-PhuS titration with apo-HemO. Biotin-labeled *prrF1*-50 (30 pM) and holo-PhuS (10 μM) was titrated with increasing concentrations of HemO (0–10 equivalents). Experiments were performed as described for Figure 2. *B*, fluorescence anisotropy (FA) of holo-PhuS titration with apo-HemO. FA was performed with a fixed concentration of holo-PhuS (1 μM) and 5′-FAM–labeled *prrF1*-50 (10 pM). The change in anisotropy was recorded as a function of apo-HemO molar equivalent until no further changes in anisotropy were recorded. *C*, as in *B* for holo-PhuS H212R. Experiments were performed in triplicate as described in the Experimental procedures section. The data were fit by converting the anisotropy, *r*, to fraction bound and plotted against HemO molar equivalents using a one-site binding model. The error is shown as the SEM.

Our previous studies characterized PhuS as a titratable regulator of heme flux through HemO (11). Based on these studies, we hypothesized that heme flux through HemO may be coupled to PhuS regulation of the *prrF1,2* operon. We tested the ability of HemO to drive the holo-PhuS to apo-PhuS conversion and subsequent DNA binding to apo-PhuS by FA and EMSA. On titration of a fixed concentration of holo-PhuS and 5′-biotinylated *prrF1*-50 with increasing concentrations of apo-HemO, we observe a lower mobility complex, consistent with apo-PhuS binding to *prrF*1-50 (Fig. 4*A*). To confirm the lower mobility complex is not because of HemO binding, we performed EMSA by titrating in increasing concentrations of apo-HemO. As shown in Fig. S3, titration of apo-HemO showed no shift in the 5′-biotinylated *prrF1-50*. Similarly, by FA analysis, titration of a fixed concentration of holo-PhuS (1 μM) and 5′-FAM-labeled *prrF1-50* (10 nM) with HemO, we observe an increase in anisotropy. The change in anisotropy when fit to a one-to-one binding site model shows saturation at ∼1:1 M equivalency of HemO to PhuS (Fig. 4*B*). Consistent with the competing equilibrium between heme transfer from holo-PhuS to HemO and apo-PhuS binding to *prrF1-50*, we observe a twofold to threefold decrease in the calculated *K*_*D*_ (140 ± 30 nM).

**Figure 5:**
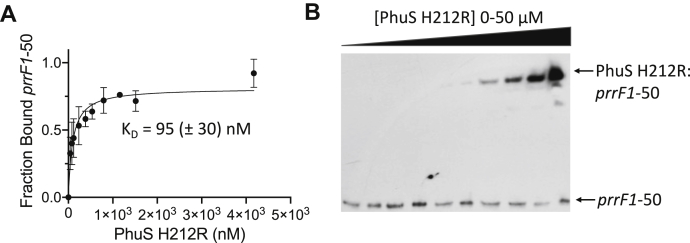
**Fluorescence anisotropy (FA) and EMSA of apo-PhuS H212R binding to *prrF1*-50.***A*, FA of apo-PhuS H212R binding to the 5′-FAM–labeled *prrF1*-50 as described for Figure 2. The data were fit by converting the anisotropy, *r*, to fraction bound and plotted against protein concentration using a one-site binding model. The error is shown as the SEM. *B*, apo-PhuS H212R binding to 5′-biotin–labeled *prrF1*-50 as described for Figure 3. All reactions contained a fixed concentration (30 pM) of labeled *prrF1*-50, and the following incubation was run on 8% acrylamide gels, transferred to a nylon membrane, and visualized by chemiluminescence.

We have previously shown that apo-PhuS undergoes a significant conformational rearrangement on heme binding (21), which likely accounts for the mutually exclusive roles in DNA binding and heme transfer. Furthermore, through site-directed mutagenesis and spectroscopic studies, we proposed a model where a conformational rearrangement on protein–protein interaction triggers a ligand switch within PhuS (from H209 to H212) prior to release to HemO. Furthermore, while the apo-PhuS H212R mutant rapidly binds heme, the rate of heme transfer to HemO is inhibited (21). We sought to determine if the altered heme binding and transfer properties of this PhuS H212R mutant influenced binding to the *prrF1* promoter. We confirmed by FA that apo-PhuS H212R binds to 5′-FAM–labeled *prrF1*-50 with a *K*_*D*_ of 90 ± 30 nM (Fig. 5*A*). Although we saw a decrease in the binding affinity and fraction bound, EMSA analysis showed a shift consistent with complex formation (Fig. 5*B*). Titration of a fixed concentration of holo-PhuS H212R and 5′-FAM-labeled *prrF1*-50 (10 nM) with HemO drives apo-PhuS binding. However, consistent with the inhibition of heme transfer, a significantly greater molar ratio of HemO to holo-PhuS H212R (5:1) is required to drive the reaction toward completion when compared with holo-PhuS WT (Fig. 4*C*).

### Heme flux through PhuS regulates PrrH but not PrrF1 levels *in vivo*

**Figure 6:**
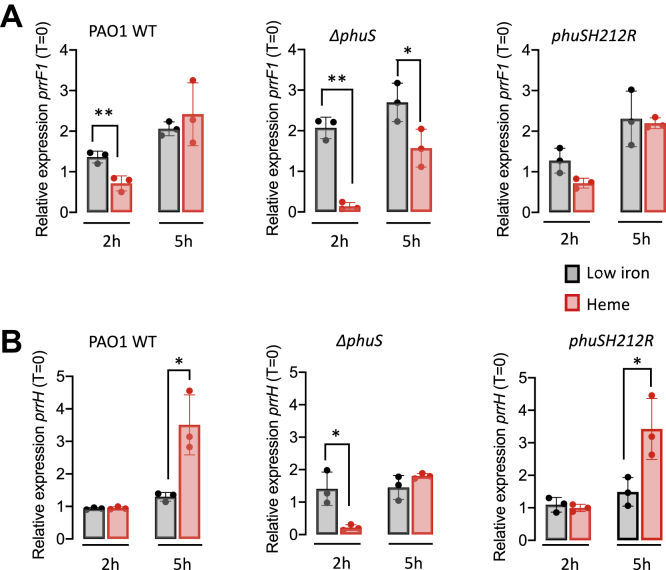
**Relative PrrF1 and PrrH sRNA levels for PAO1 WT, Δ*phuS*, and the *phuSH212R* allelic strain in iron-deplete or heme-supplemented conditions.***A*, PrrF1 relative sRNA levels. *B*, PrrH relative sRNA levels. mRNA isolated at 0, 2, and 5 h following growth in either iron-deplete M9 or M9 supplemented with 1 μM heme. mRNA values represent the mean from three biological experiments, each performed in triplicate and normalized to 0 h. *Gray* shaded bars represent iron-deplete conditions; *red* shaded bars represent heme-supplemented conditions. *Error bars* represent the standard deviation from three independent experiments performed in triplicate. *p* values as determined by two-tailed Student's *t* test comparing values upon heme supplementation to iron-deplete conditions at the same time point, where ∗*p* < 0.05.

To assess the role of PhuS in transcriptional regulation of *prrF1* and/or *prrH*, we performed qPCR analysis of PAO1 WT, a *phuS* knockout (Δ*phuS*) and the *phuSH212R* allelic strain in low iron- and heme-supplemented conditions. All the strains utilized had similar growth rates in iron-deplete or heme-supplemented conditions (Fig. S4). Through a combination of isotopic ^13^C-heme uptake followed by LC–MS/MS and inductively coupled plasma—MS, we have previously shown that 1 μM of heme-supplemented cultures deplete the exogenous heme by 7 to 8 h, resulting in Fur repression (13, 22, 23). Therefore, we analyzed the relative PrrF and PrrH levels at 2 and 5 h where heme flux through PhuS is maximal and prior to Fur repression. The PrrF probe (Table S2) detects PrrF, PrrF2, and PrrH sRNAs owing to the similarity and overlap in sequences. In contrast, the PrrH probe comprising the unique intergenic sequence between *prrF1* and *prrF2* detects PrrH specifically (Table S2). Given the previously reported low abundance of PrrH compared with PrrF1 and PrrF2 (17), the contribution of PrrH to the relative RNA levels measured with the PrrF probe is negligible. In iron-deplete conditions, we see an approximately twofold increase in PrrF at 5 h consistent with iron deprivation (Fig. 6*A*; *left panel*). However, in heme-supplemented conditions at 2 h, we observe an initial approximately twofold decrease in the relative PrrF levels. Nevertheless, at 5 h, the relative expression of PrrF on heme supplementation is identical to that in low iron conditions (Fig. 6*A*; *left panel*). We have previously observed a similar heme-dependent decrease in relative RNA levels at the early 2 h time point for Fur-regulated genes within the *has* and *phu* operons (13, 22). We attribute this decrease to an initial effect of the influx of heme or iron. In contrast to PrrF, PrrH levels show no increase over time in low iron (Fig. 6*B*; *right panel*). However, in heme-supplemented conditions, we see a significant threefold to fourfold increase in PrrH levels at 5 h (Fig. 6*B*; *right panel*). Furthermore, at the 2 h time point, we do not observe the initial decrease in the relative expression of PrrH as seen for PrrF. Therefore, in contrast to PrrF, which is under transcriptional regulation of Fur, PrrH is not iron regulated but is subject to positive regulation by heme.

To confirm that the heme-dependent increase in PrrH expression is indeed mediated by PhuS, we performed qPCR analysis on the Δ*phuS* strain. In low iron, we observe an approximately twofold increase in PrrF expression at the earlier 2 h time point in the Δ*phuS* strain (Fig. 6*A*; *middle panel*). Interestingly, the decrease in relative expression of PrrF in heme-supplemented conditions compared with low iron is significantly enhanced in the Δ*phuS* strain compared with PAO1 WT (Fig. 6*A*; *middle panel*). On heme supplementation, we observe a loss in the heme-dependent regulation of PrrH in the Δ*phuS* strain (Fig. 6*B*; *middle panel*). Deletion of PhuS not only leads to a loss in the heme-dependent increase in PrrH expression but also appears to increase the iron effect over the *prrF1* promoter. Given the partial overlapping binding sites of PhuS and Fur, it is possible that the absence of PhuS may allow for increased access to the Fur box and enhanced repression of PrrF in the Δ*phuS* knockout. Although not statistically significant, in low iron, the loss of PhuS appears to show slightly elevated levels of PrrF relative to PAO1 WT (Fig. 6*A*). Taken together, the data suggest that PhuS binding as a function of heme status regulates the relative expression of PrrF1 and PrrH through modulation of Fur binding.

Based on the increased molar ratio of HemO required to drive PhuS H212R binding to the *prrF1* promoter *in vitro*, we sought to determine if such changes in heme binding and transfer influence PrrH expression. The relative expression profiles of both PrrF1 and PrrH in the *phuSH212R* allelic strain were very similar to that of PAO1 WT in both low iron and heme (Fig. 6, *A* and *B*; *right panels*). Taken together, the decrease in binding affinity and shift in the molar ratio of HemO required to drive apo-PhuS DNA binding *in vitro* are not significant enough to disrupt the PhuS–HemO equilibrium *in vivo*. Future studies with variants disrupting either heme transfer or the PhuS–HemO protein–protein interaction will be undertaken to investigate the role of extracellular heme flux on PrrF and PrrH expression.

## Discussion

Iron acquisition and homeostasis are critical for *P. aeruginosa* survival and pathogenesis. Bacterial iron homeostasis is maintained by either repressing the expression of iron-uptake systems in iron-replete conditions or by decreasing the levels of iron-containing proteins in iron-limiting conditions. The latter function is most often mediated at the post-transcriptional level by iron-responsive sRNAs, which in many cases also regulate virulence traits (14, 24, 25, 26, 27). In *P. aeruginosa*, the PrrF sRNAs play a role in numerous other processes, including twitching motility, quorum sensing molecule biosynthesis, and biofilm formation (16, 18). Specific PrrF targets include the iron-containing proteins superoxide dismutase (*sodB*), succinate dehydrogenase (*sdh*), and a heme-containing catalase (*katG*) (14). PrrF also indirectly promotes the production of the *Pseudomonas* quinolone signal (PQS) by repressing *antR*, an activator of genes required for degradation of the PQS biosynthetic precursor anthranilate (15). Therefore, the PrrF sRNAs contribute to virulence through both the iron-sparing response and the activation of PQS-regulated virulence factors. However, the regulatory mechanisms by which *P. aeruginosa* adapts to a particular iron source are not as well understood. For example, in chronic infection, *P. aeruginosa* decreases its reliance on siderophores, while simultaneously increasing reliance on heme (28, 29). This increased dependence on heme coincides with the upregulation of the Phu heme uptake system.

**Figure 7:**
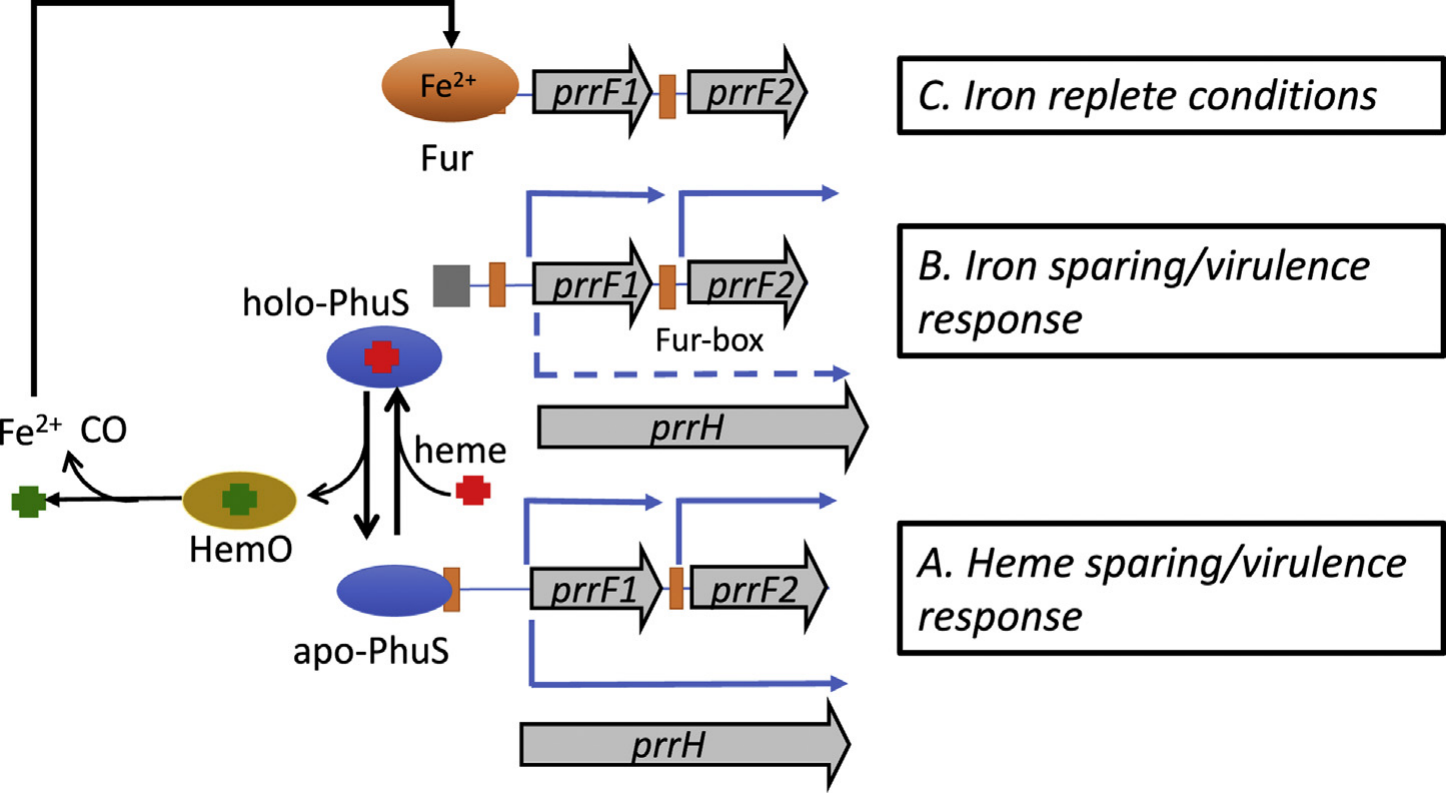
**Proposed model for the heme-dependent modulation of PrrF and PrrH expression by PhuS**.

The heme uptake systems like their siderophore counterparts are globally regulated by the master regulator Fur but must also have additional levels of regulation that allow for a coordinated transcriptional response to heme. Some years ago, it was reported that the tandem arrangement of *prrF1* and *prrF2* allows for expression of a longer heme-responsive sRNA PrrH, predicted to affect the expression of genes related to heme homeostasis (17). Given the genetic link between the *prrF1,F2* locus and *phuS*, we hypothesized that heme flux through PhuS may play a role in integrating heme metabolism into the sRNA regulatory network. Herein, we show that apo-PhuS specifically binds within the *prrF1* promoter and modulates the expression of PrrF and PrrH as a function of extracellular heme flux. We propose a model whereby the equilibrium between apo-PhuS and holo-PhuS modulates the relative expression of PrrH (Fig. 7). In this model, under low iron conditions, the equilibrium shift to apo-PhuS leads to increase in the relative expression of PrrH on binding and reorganization of the *prrF1* promoter (Fig. 7*A*). Active heme uptake shifts the equilibrium toward holo-PhuS downregulating the relative levels of PrrH compared with PrrF1 and/or PrrF2 (Fig. 7*B*). The eventual increase in intracellular iron levels as a function of heme utilization leads to Fur repression of the *prrF1,2* operon (Fig. 7*C*). The fact that optimal apo-PhuS binding includes the Fur box (Fig. 2*C*) but has no affinity for the Fur box alone (Fig. 2*C*) suggests that the PhuS and Fur binding sites are not mutually exclusive but may be antagonistic. This is supported in part by the qPCR data, where in iron-limiting conditions, the absence of PhuS increases the relative levels of PrrF compared with PAO1 WT (Fig. 6*A*). In contrast, in heme-supplemented conditions, the initial iron-dependent repression of PrrF is significantly increased, presumably a consequence of greater access of Fur to the Fur box in the absence of apo-PhuS (Fig. 6*A*). Similarly, the increased Fur repression and loss of PhuS also leads to a decrease in the relative expression of PrrH. These studies suggest that apo-PhuS binding to the *prrF1* promoter as a function of Fur antagonism allows for a coordinated iron and heme transcriptional response. A previous study has shown that the *prrF* operon requires extended upstream sequence for full promoter activity (18), a common feature of promoters that bind multiple transcription factors as higher order oligomers and show promiscuous DNA shape-dependent binding at sites distant from the transcriptional start site (30, 31). The Fur proteins themselves are known to oligomerize in a metal-dependent manner and bind to promoters at multiple sites causing DNA looping (31, 32, 33). Similarly, the PhuS homolog ShuS was shown by atomic force microscopy techniques to form oligomeric complexes condensing the DNA (20). The fact that the PhuS-protected fragment (Fig. 1*B*) is ∼200 bp is consistent with PhuS having similar nucleoid-associated protein-like properties that include oligomerization and promiscuous binding specificity. It is not clear at the present time how modulation of PhuS and Fur binding to the *prrF1* promoter allows for remodeling of the DNA structure or read through of the *prrF1* transcriptional terminator required for PrrH expression. Interestingly, upstream of the *prrF2* Fur box is an AlgR-binding site that has been shown to directly and indirectly regulate pyoverdine biosynthesis (34). The AlgR transcriptional regulator is part of the *algZR* two-component sensor system that regulates alginate as well as several virulence factors, including type IV pillus, rhamnolipid production, Rhl quorum sensing system, and biofilm formation (35, 36, 37, 38, 39). It is possible given the proximity of the *prrF1* and *prrF2* promoters that are separated by only 95 bps that short-range DNA interactions driven by higher order multimers or overlapping interactions of the transcriptional regulators allow for differential expression of PrrF1, PrrF2, and/or PrrH. A precedent for such a mechanism has been characterized in *Helicobacter pylori* where oligomerization and DNA condensation by Fur and its antagonism by the Ni-dependent NikR allows for integration of metal homeostasis and acid acclimation (31). A more extensive analysis by DNAse I footprinting and expression analysis under different conditions will determine if these transcriptional regulators physically interact and coordinate transcriptional regulation *via* structural changes within the *prrF1* and *prrF2* promoters.

Differential regulation over the *prrF1* and *prrF2* promoters provides a mechanism by which the relative expression levels of PrrF1, PrrF2, and PrrH may ultimately determine the distinct target profiles of the sRNAs. This is especially true of PrrF1 and PrrF2 that have almost identical sequences. Interestingly, in PAO1, *algR* is cotranscribed with the *hemCD* genes providing a link to intracellular heme biosynthesis (40). Furthermore, the AlgR regulation of pyoverdine provides a link between heme biosynthesis and iron homeostasis (34). While specific targets of PrrH are not as well characterized as the identification of potential PrrH targets such as *vreE*, a regulator of virulence, the heme-d_1_ biosynthesis gene *nirL*. The *nir* gene cluster encodes genes for the synthesis of heme d_1_ and branches from the central heme biosynthesis pathway at uroporphyrinogen III. Thus, PrrH repression of heme d_1_ biosynthesis may prioritize heme precursors to produce the more abundant heme b. Therefore, PhuS-dependent modulation of PrrH may further allow for integration of iron and heme homeostasis with the virulence networks of *P. aeruginosa* (16, 17). Indeed, the Δ*prrF1,F2* mutant is defective for both heme and iron homeostasis and is attenuated for virulence in an acute mouse lung infection model (18). Therefore, it is reasonable to suggest that the modulation of the iron-dependent PrrF/PrrH network by PhuS and AlgR plays a role in unifying intracellular iron and heme homeostasis as well as virulence traits required for infection. The ability to rapidly respond and adapt to heme as an iron source is likely to provide a competitive advantage in the host. As previously mentioned, in chronic infection, *P. aeruginosa* adapts over time to utilize heme as an iron source *via* the Phu system, while decreasing its reliance on siderophore systems (28). The fact that the tandem arrangement of the *prrF* genes and the presence of *phuS* are genetically linked and found only in pathogenic *P. aeruginosa* highlights the significance of the iron- and heme-dependent sRNAs in this adaptive response (17). Furthermore, the detection of both PrrF and PrrH in infected murine lungs as well as sputum from patients with cystic fibrosis further signifies a role for these sRNAs during infection (18).

In summary, we have identified PhuS as a heme-dependent transcriptional regulator of PrrH expression in addition to its role in regulating extracellular heme flux through HemO. This is also the first report in *P. aeruginosa* of a regulatory link between extracellular heme metabolism and the iron- and heme-dependent sRNAs. This dual function of PhuS is central to integrating extracellular heme utilization into the PrrF/PrrH sRNA regulatory network critical for *P. aeruginosa* adaptation and virulence within the host. Based on these preliminary studies, PhuS offers an advantage as a potential antimicrobial target; it is found only in pathogenic *P. aeruginosa* strains, and its dual function in pathways central to survival and pathogenesis in the host is potentially advantageous in slowing resistance development. A more complete understanding of the molecular mechanisms by which PhuS regulates a coordinated transcriptional response from the *prrF1* promoter will be critical in the development of novel strategies to target iron homeostasis and virulence.

## Experimental procedures

### Bacterial strains and growth conditions

Bacterial strains and plasmids used in this study are listed in Table S1, and oligonucleotide primers and probes used in this study are listed in Table S2. All primers and probes used in this study were purchased from Integrated DNA Technology. *Escherichia coli* strains were routinely grown in LB broth (American Bioanalytical) or on LB agar plates. *P. aeruginosa* strains were freshly streaked and maintained on *Pseudomonas* isolation agar (BD Biosciences). All strains were stored at −80 °C in LB with 20% glycerol. The iron levels in M9 medium (Nalgene) were determined by inductively coupled plasma–MS to be less than 1 nM. For qPCR, singly isolated colonies from each *Pseudomonas* strain were picked, inoculated into 10 ml of LB broth, and grown overnight at 37 °C with shaking (210 rpm). The bacteria were then harvested and washed in 10 ml of M9 minimal medium. Following centrifugation, the bacterial pellet was resuspended in 10 ml of M9 medium and used to inoculate 50 ml of fresh M9 iron-deplete medium to a starting *A*_600_ of 0.04. Cultures were grown at 37 °C with shaking for 3 h before the addition of supplements (0 h) and incubated for a further 6 h. When required, antibiotics were used at the following final concentrations: tetracycline 10 and 150 μg ml^−1^ for *E. coli* and *P. aeruginosa*, respectively. When required, ampicillin was used at a final concentration of 100 μg/ml.

### Construction of the *P. aeruginosa phuSH212R* allelic strain

*phuSH212R* was obtained by allelic exchange as previously described (41), using the parental strain PAO1 Δ*phuS* (42). Briefly, a 2.9-kb *phuS* gene fragment including upstream and downstream sequences was PCR amplified from the chromosomal DNA of *P. aeruginosa* PAO1 using primer pairs *Pst*I-5′PhuS-F and *Hind*III-3′PhuS-R. The amplified fragment was cloned into pUC18, resulting in pUC18 to 5′-PhuS-3′. The mutant allele *phuSH212R* was obtained following digestion of plasmid pET21*phuS*H212R (21) with *Nru*I and *Stu*I and subcloned into *Nru*I- and *Stu*I-digested pUC18 to 5′-PhuS-3′, replacing the WT allele. The new construct pUC18 to 5′-*phuS*H212R-3′ was confirmed by sequencing (Eurofins MWG Operon). The insert including *phuS*H212R plus the 5′ and 3′ flanking regions was purified by *PstI*-*Hind*III digestion and ligated into the counter-selective suicide plasmid pEX18Tc (41). Finally, plasmid pEX18Tc-5′–*phuS*H212R-3′ was transferred into *P. aeruginosa* Δ*phuS* by conjugation. A double event of homologous recombination followed by selection on *Pseudomonas* isolation agar plates containing 5% sucrose resulted in chromosomal integration of *phuS*H212R, replacing the parental allele Δ*phuS*. PCR and sequencing analysis were used to verify the allelic exchange process.

### Expression and purification of apo-PhuS and PhuSH212R

Protein expression was performed as previously reported with slight modification (10, 43). The PhuS or PhuSH212R mutant lysate was applied to a Sepharose-G column (GE Life Sciences) equilibrated with 20 mM Tris–HCl (pH 8.0) and washed with five column volumes of the same buffer. The column was further washed with 10 column volumes of 20 mM Tris (pH 8.0) containing 20 mM NaCl, and the PhuS protein was eluted with a linear gradient of 50 to 500 mM NaCl. Eluted fractions were analyzed by SDS-PAGE, and the peak fractions were pooled and dialyzed against 4 l of 20 mM Tris (pH 8.0) containing 100 mM NaCl. The protein was concentrated in a Pierce Protein Concentrator (30 K) (Thermo Fisher Scientific) and purified to homogeneity on an AKTA FPLC system fitted with a 26/60 Superdex 200 pg size exclusion column (GE Life Sciences) equilibrated with 20 mM Tris (pH 8.0) containing 100 mM NaCl. Peak fractions as judged by the A_280_ were subjected to SDS-PAGE, and the pure fractions were pooled, concentrated (10 mg/ml), and stored at −80 °C until further use.

The histidine-tagged protein PhuS-His_6_ was expressed as for the non–His-tagged PhuS. The lysate following removal of the cell debris was applied directly to a Ni-NTA–agarose (Thermo Fisher Scientific) column (1 × 5 ml) previously equilibrated with 20 mM Tris (pH 8.0) containing 0.5 M NaCl and 5 mM imidazole. The column was washed with 10 volumes of equilibration buffer, followed by 10 volumes of wash buffer (20 mM Tris, pH 8.0, containing 0.5 M NaCl and 60 mM imidazole), and the protein eluted in 20 mM Tris (pH 8.0) containing 0.25 M NaCl and 500 mM imidazole. The purified protein was exchanged by dialysis into 20 mM Tris (pH 8.0) containing 100 mM NaCl concentrated (10 mg/ml) and stored at −80 °C until further use.

Heme solutions were prepared in 0.1 N NaOH, and the pH adjusted with the identical buffer was used to prepare the PhuS protein samples. Heme loading of the purified PhuS protein was carried out by addition of a 1.5:1 ratio of heme to protein. Excess heme was removed over a Sephadex G-50 column (GE Life Sciences) equilibrated with 20 mM Tris (pH 8.0). All buffered heme solutions were used within 20 min of preparation. Heme stock solution concentrations and the stoichiometry of the final holo-PhuS complexes were determined by pyridine hemochrome as previously described (44).

### Expression and purification of HemO

HemO was purified as previously reported with slight modification (12). HemO lysate was applied to a Q-Sepharose Fast Flow column (2.5 × 6 cm) (GE Life Sciences) equilibrated with 20 mM Tris (pH 8.0 at 4 °C) and 100 mM NaCl. Protein was eluted with a 20 mM Tris (pH 8.0 at 4 °C) and 100 to 500 mM NaCl gradient. Peak protein fractions were determined *via* SDS-PAGE and were pooled, concentrated, and dialyzed against 20 mM Tris buffer (pH 8.0) and 100 mM NaCl at 4 °C. The protein (5–6 ml) was further purified by FPLC over a 26/60 Superdex 200 pg size exclusion column (GE Life Sciences) equilibrated with 20 mM Tris (pH 8.0) containing 100 mM NaCl. Peak fractions as judged by the A_280_ were subjected to SDS-PAGE, and the pure fractions were pooled, concentrated, (10 mg/ml), and stored at −80 °C until further use.

### Expression and purification of *P. aeruginosa* Fur

The Fur protein was expressed and purified as previously described (45). The Fur lysate, conjugated with glutathione-*S*-transferase (GST), was placed in a glutathione super-flow column (Clontech), equilibrated with 20 mM Tris–HCl (pH 8.0), and washed with five column volumes of the same buffer. The protein was eluted with 50 mM Tris–HCl (pH 8.0) and 33 mM glutathione, and eluted fractions were analyzed by native-PAGE. Fractions containing GST-*pa*Fur were cleaved using a Thrombin CleanCleave kit (Sigma–Aldrich). Briefly, an aliquot of thrombin–agarose resin (50% v/v) was mixed with 1 mg of GST-*pa*Fur and 100 μl of 10× cleavage buffer. The mixture was incubated at 37 °C for 3 h while collecting 10 μl aliquots at every hour. Fractions were measured by the A_280_ and checked for purity *via* SDS-PAGE. The fully cleaved protein was pooled and exchanged by dialysis in 20 mM Tris–HCl (pH 8.0), concentrated (10 mg/ml), and stored in −80 °C until further use.

### ChIP–PCR

A single isolated colony of *Pseudomonas* PAO1 or Δ*phuS* strains was used to inoculated 10 ml of LB broth and grown overnight at 37 °C. The bacteria were then harvested and resuspended in 2 ml of M9 minimal medium. The resuspended cultures were used to inoculate 25 ml of fresh M9 low-iron medium to a starting *A*_600_ of 0.04. Cultures were grown at 37 °C with shaking for 5 h. Cells were harvested at 7000*g* for 3 min (25 °C) and resuspended in 2 ml of M9 minimal media that were used to inoculate 25 ml of M9 medium to a starting *A*_600_ of 0.04. Cultures were grown for 3 h in iron-limiting conditions before the addition of 0.5 μM heme. Following an additional 2 h, cells were harvested at 7000*g* for 10 min and resuspended in 2 ml of PBS. The aliquots were treated with formaldehyde to 1% (v/v). The cells were gently agitated at room temperature for 10 min, and then the crosslinking was quenched with glycine to a final concentration of 10 mg/ml. Cells were then gently shaken at 4 °C for 30 min, centrifuged, and washed twice with PBS. Finally, cells were resuspended in 2 ml of lysis buffer (100 mM Tris [pH 8.0] containing 300 mM NaCl, 10 mM EDTA, 0.1 mM PMSF, and 50 μg/ml lysozyme), mixed 10 min at 4 °C, and then sonicated (50 s with 5 s pulse and 1 min pause at 80% amplitude) before its centrifugation to remove cell debris. Cell extracts were aliquoted into 1-ml volumes and frozen at −80 °C until further use. Magnetic beads conjugated with IgG Protein A/G (New England Biolabs) were preblocked with 0.5 mg/ml of sonicated salmon sperm DNA (Thermo Scientific) and bovine serum albumin (BSA; Sigma–Aldrich) and washed with 100 mM Tris (pH 8.0) containing 300 mM NaCl to create a slurry. Lysates were precleared with 50 μl of the bead slurry per 500 μl of cell lysate and incubated with gentle agitation at room temperature for 1 h, followed by centrifugation for 5 min at 2500*g* (4 °C). The supernatant was collected, and total protein concentration was measured *via* the bicinchoninic assay (BioRad). Supernatants were split into 2 × 500 μl samples and 2 μl of anti-PhuS serum was added to one of the samples. Both samples were then incubated overnight at 4 °C with rotation. Antibodies were obtained from Covance Custom Antibodies and generated from purified proteins supplied by our laboratory. Antibody specificity and sensitivity was previously determined with the respective purified proteins. Washed-bead slurry of 100 μl was added to all samples and mixed for 30 min at 4 °C, and the samples were centrifuged as before. Supernatant from the negative control was saved to use as input DNA. The protein–DNA complex was washed with 1× PBS several times and eluted with 0.1 to 0.2 M of glycine–HCl buffer (pH 2.5–3.0). The elution was neutralized by addition of 1 M Tris buffer (pH 8). The protein–DNA complex was treated with 1 U/μl DNase I (Novagen) to digest nonspecific DNA. The protein–DNA complex was uncrosslinked by adding 0.2 M of NaCl and incubating overnight at 65 °C. The remaining DNA was purified using the QIAquick PCR Purification kit (Qiagen) following the manufacturer's instructions. Specific primers designed within the *prrF1,F2* promoter region were used to amplify the isolated fragment (Table S2). PCR products were analyzed *via* agarose electrophoresis and visualized under UV light in an AlphaImager gel doc (Protein Simple). PCR-amplified products were sequenced to confirm specificity.

Pull downs with purified PhuS-His_6_ and gDNA of *P. aeruginosa* were performed as follows: gDNA (3 μg) was digested by *Hpa*II (New England Biolabs) at 37 °C for 1 h. The digested samples were loaded on an agarose gel to detect and purify fragments ranging from 100 to 500 bps using the Monarch DNA Gel Extraction Kit (New England Biolabs). The fragmented gDNA was incubated for 1 h with 10 μM PhuS in 50 mM Tris–HCl (pH 8.0), 100 mM NaCl, 1 mM PMSF, and one complete mini protease inhibitor tablet (Roche) on a tabletop roto shaker (Scientific Industries). Ni-NTA resin of 100 μl (Themo Fisher Scientific) was added to the mixture and incubated for 5 min at 4 °C. The resin was centrifuged at maximum speed (∼10,000*g*) for 1 min and 3 times with 50 mM Tris–HCl (pH 8.0) containing 100 mM NaCl. PhuS-His_6_ was eluted from the resin with 50 mM Tris–HCl (pH 8.0) containing 100 mM NaCl and 250 mM imidazole. The eluant was treated with DNase I (1 U/μl) to digest nonspecific DNA, and the bound DNA was purified from the complex *via* PCR Qiagen Kit (Qiagen). The purified DNA was amplified with primer sets specific to the *prrF1,F2* promoter (Table S2) analyzed *via* agarose electrophoresis and sequenced as described previously.

### EMSA

DNA fragments for EMSA experiments were obtained by annealing the 5′-biotin–labeled oligonucleotides (Table S2). Sense and antisense oligonucleotides were annealed by mixing a 1:1 ratio, incubating at 95 °C for 5 min followed by cooling down to room temperature. DNA was cleaned up with the QIAquick Nucleotide Removal Kit (Qiagen), and the concentration measured by UV absorption at 260 nm in a NanoDrop 2000c Spectrophotometer (Thermo Scientific). All protein oligonucleotide-binding reactions were assayed in 100 mM Tris (pH 8.0) containing 500 mM NaCl, 10% glycerol, and 10 ng/ml salmon sperm DNA. Apo-PhuS protein concentrations ranged from 0.1 to 25 μM. For the HemO titration reactions, holo-PhuS was fixed at 10 μM. HemO protein concentrations were varied across a range of 0 to 12 M equivalents. All reactions were incubated at 37 °C for 20 min before the addition of the biotinylated probe. For all reactions, the oligonucleotides were used at a fixed concentration of 30 pM in a final volume of 10 μl. The reactions were incubated for a further 20 min at 37 °C and analyzed on an 8% Tris–glycine acrylamide native gel. The gel was prerun in 1× Tris–glycine buffer (pH 8.3) for 1 h at 200 V 4 °C and run 2 h at same voltage. DNA was transferred to positively charged nylon membrane (BrightStar-Plus Positively Charged Nylon Membranes; Invitrogen) using a Semidry Electroblotting System (Thermo Scientific) with 1× Tris–glycine (pH 8.3) for 30 min at 300 mA. Membranes were washed in 2× saline-sodium citrate buffer (Thermo Scientific), for 5 min at room temperature, and DNA immobilized by UV crosslinking. The position of the nucleic acids was visualized by chemiluminescent detection using the Chemiluminescent Nucleic Acid Detection Module (Thermo Scientific) following manufacturer's instructions and exposed to X-ray film (Amersham hyperfilm ECL; Amersham).

### FA

Binding of Fur or apo-PhuS to the 5′-FAM oligonucleotides (Table S2) was assessed using FA as previously described (20). Briefly, 5′-FAM oligonucleotides designed within the *prrF1/prrF2* promoter region (Table S2) were analyzed by UV–visible spectroscopy to quantify the percentage of fluorescein tag. Double-stranded oligonucleotides were obtained by combining a 1:1 ratio of the 5′-labeled sense and antisense oligonucleotides in deionized H_2_O. To facilitate annealing, mixtures were heated to 95 °C, 5 min, and cooled down to room temperature. The labeled double-stranded oligonucleotides were stored at −80 °C until further use. In a quartz cuvette, 10 nM of 5′-FAM oligonucleotide was diluted in 20 mM Tris–HCl (pH 8.0), containing 100 mM NaCl, and 0.05 mg/ml BSA in a final volume of 500 μl. For runs with Fur, 10 nM of 5′-FAM oligonucleotide was diluted in 10 mM bis–Tris borate (pH 7.5), 40 mM KCl, 0.1 mM MnSO_4_, 0.1 mg/ml BSA, and 10% glycerol. All measurements were performed on a K2 spectrofluorometer (ISS) configured in the L-format, with excitation/emission wavelengths and band widths of 495 and 2 nm and 519 and 1 nm, respectively. A measurement of the maximum anisotropy was performed on the 5′-FAM oligonucleotide, and the change in anisotropy was measured as a function of increasing concentrations of apo-PhuS or Fur. The addition of protein continued until no further change in anisotropy was observed. The data were fit by converting the anisotropy, *r*, to fraction bound, *F*_bound_ (the fraction of protein bound to the oligonucleotide at a given DNA concentration), using the following equation:

$$F_{\text{bound}}=\frac{r-r_{\text{free}}}{\left(r_{\text{bound}}-r\right)Q+\left(r-r_{\text{free}}\right)}$$

where *r*_free_ is the anisotropy of the fluorescein-labeled oligonucleotide and *r*_bound_ is the anisotropy of the oligonucleotide–protein complex at saturation. The quantum yield designated as *Q* is calculated from the changes in fluorescence intensity that occurs over the course of the experiment (*I*_bound_/*I*_free_). *F*_bound_ was then plotted against the protein concentration using a one-site binding model:

$$P+D↔PD\frac{\left[P\right]\left[D\right]}{\left[PD\right]}=K_{d}$$

$$F_{\text{bound}}=\left\{P_{\text{total}}+D_{\text{total}}+K_{d}\right\}-\left[{\left(\left(\sqrt[{2}]{P_{\text{total}}+D_{\text{total}}+K_{d}}\right)-4P_{\text{total}}D_{\text{total}}\right)}_{\frac{1}{2}}\right]/2D_{\text{total}}$$

where *P* is the protein concentration and *D* is the DNA concentration. All concentrations and fluorescence changes were done in triplicate and corrected for volume changes. For the apo-HemO titrations, the experiments were performed as described previously with a fixed concentration of 5′-FAM–labeled *prrF*1-50 (10 nM) and holo-PhuS (1 μM). The change in anisotropy was recorded on addition of increasing concentrations of apo-HemO and converted to fraction bound and plotted against HemO molar equivalents.

## Data availability

All data are contained within the article.

## Conflict of interest

The authors declare that they have no conflicts of interest with the contents of this article.
