# Supplementary material for: The heme-binding protein PhuS transcriptionally regulates the Pseudomonas aeruginosa tandem sRNA prrF1,F2 locus
Source: J Biol Chem. 2021 Jan 9;296:100275. doi: 10.1016/j.jbc.2021.100275 (PMC7948967; doi:10.1016/j.jbc.2021.100275)
Supplement: Supplemental Figures and Tables [file mmc1.pdf]

Supporting Information

The heme binding protein PhuS transcriptionally regulates the *Pseudomonas aeruginosa* tandem sRNA *prrF1,F2* locus

Tyree Wilson, Susana Mouriño and Angela Wilks*

Department of Pharmaceutical Sciences, School of Pharmacy, University of Maryland, Baltimore, MD 21201.

<u>Table of Contents</u>

Table S1. Strains and plasmids used in this study.

Table S2. Oligonucleotide primers and probes used in this study.

Figure S1. PhuS-His_6 cross-linking and pull down of PAO1 WT genomic DNA .

Figure S2. Change in anisotropy (*r*) on PhuS binding to the *prrF2* and *prrF1*(No Fur) promoters.

Figure S3. EMSA of apo- HemO binding to *prrF1-50.*

Figure S4. Growth curves for PAO1 WT and Δ*phuS* strains in iron-deplete and heme supplemented media.

Table S1. Strains and plasmids used in this study.

Strains	Relevent genotype or description	Source of Reference
E. coli		
BL21 (DE3)	F¯ ompT hsdSB (rB¯ mB¯) gal dcm (DE3)	Stratagene
DH5α	fhuA2 lac(del)U169 phoA glnV44 Φ80' lacZ(del)M15 gyrA96 recA1 relA1 endA1 thi-1 hsdR17	Thermo Fisher Scientific
P. aeruginosa		
PAO1	Wild type	(1)
Δ*phuS*	Chromosomal in frame *phuS* deletion in PAO1	(2)
phuSH212R	Chromosomal in frame *phuSH212R* allele in PAO1	This Study
**Plasmids**		
pUC18	Amp^R; Subcloning vector	Thermo Fisher
pET21a-*phuS*	Amp^R, *phuS* wild-type cloned into pET21a plasmid by T4 DNA Ligase	(3)
pET21a-*phuSH212R*	Amp^R, *phuS* mutant allele cloned into pET21a plasmid by T4 DNA Ligase	(4)
pGST-*paFur*	Amp^R, *fur* wild-type cloned into pGEX-2T plasmid by T4 DNA Ligase	(5)
pET21a-*hemO*	Amp^R, *hemO* wild-type cloned into pET21a plasmid by T4 DNA Ligase	(6)
pEX18Tc	Tc^R, allelic replacement vector	(7)

Table S2. Primers and Probes used in this study.

**Name**	**Sequence (5' to 3')**
**Primers and probes**	
PR1	5'-ACTGCGTGGGTCTCTCAG-3'
PF1	5'-GAATCGCCCATAGCCTGATCG-3'
PF2	5'-CTGCTTAACCGGGAAGTGAC-3'
PF3	5'-CTCGCGACTAGCTAGCAGAA-3'
hasR-F	5'-CGTGGCGTCGAGTACCAG-3'
hasR-R	5'-GGTCTTCGAACAGAAGTCGTTG-3'
PstI-5'PhuS-F	5'-CGCTGCAGCATAGGCGCTCTTCTGGTCGG-3'
HindIII-3'PhuS-R	5'-GCAAGCTTGGCGGCTTCCGTACTCAGCG-3'
prrF1-50 F	5'-(6FAM)(Biosg)/AGCAGAAAAGTTTGGCGAAAGCGTTTGACATGGAAATGAGAATCATTATT-3'
prrF1-50 R	5'-Biosg/AATAATGATTCTCATTTCCATGTCAAACGCTTTCGCCAAACTTTTCTGCT-3'
prrF1-50 (No Fur) F	5'-6FAM/ATTCCAGAGGGCTCGCGACTAGCTAGCAGAAAAGTTTGGCGAAAGCGTTT-3'
prrF1-50 (No Fur) R	5'-AAACGCTTTCGCCAAACTTTTCTGCTAGCTAGTCGCGAGCCCTCTGGAAT-3'
prrF1-30 F	5'-6FAM/GACATGGAAATGAGAATCATTATTATGTCA-3'
prrF1-30 R	5'-TGACATAATAATGATTCTCATTTCCATGTC-3'
prrF2-50*(Fur)* F	5'-6FAM/ATGAGAACCGGCTTGACCTGATAATGAGAATAGTTATTATTACACCAACT-3'
prrF2-50*(Fur)* R	5'-AGTTGGTGTAATAATAACTATTCTCATTATCAGGTCAAGCCGGTTCTCAT-3'
prrF2-50 *(AlgR)* F	5'-6FAM/GCCTGCGATTCGGCCGGAGACGACCGTTCATCGGCTGGCGATGGAATGAA-3'
prrF2-50 *(AlgR)* R	5'-AGTTGGTGTAATAATAACTATTCTCATTATCAGGTCAAGCCGGTTCTCAT-3'
**RT-qPCR Primers and Probes**	
qPCR-*prrH* Probe	5'-6FAM/CTGGCGATGGAATGAATGAG/BHQ-1-3'
qPCR-*prrH* F	5'-ATTCGGCCGGAGACGAC-3'
qPCR-*prrH* R	5'-CGACCAGTTGGTGTAAT-3'
qPCR-*prrF* Probe	5'-6FAM/TAAGCTGAGAGACCCACGCAG/BHQ-1-3'
qPCR-*prrF* F	5'-AACTGGTCGCGAGATCAGC-3'
qPCR-*prrF* R	5'-CCGTGATTAGCCTGATGAGGAG-3'
qPCR-23S Probe	5'-6FAM/GTAAGTGACGCGGTAGAGGAGCGTTCTGTA/BHQ-1-3'
qPCR-23S F	5'-GGGCTCAAACCACACACC-3'
qPCR-23S R	5'-GCTTCTCAACTCACCTTCACAG-3'

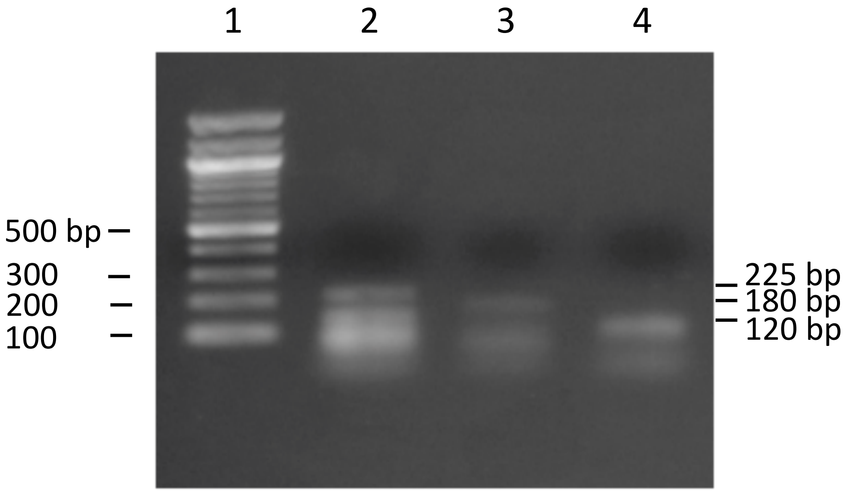

Figure S1. PhuS-His$_6$ cross-linking and pull down of PAO1 WT genomic DNA. DNA fragments obtained following crosslinking of PhuS-His$_6$ to genomic DNA and pull-down with Ni-NTA agarose. 1. Molecular weight markers as shown, 2. 225 bp amplified with primers PF1 and PR1, 3. 180 bp fragment amplified with PF2 and PR1, 4. 120 bp fragment amplified with primers PF3 and PR1. PCR fragments were visualized on 1% agarose with ethidium bromide staining.

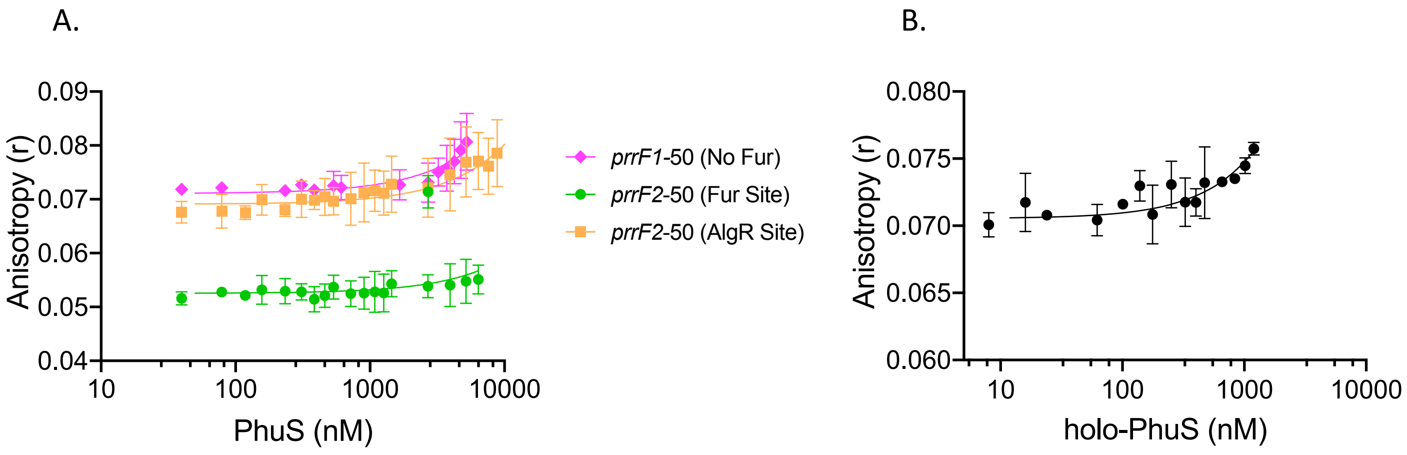

Figure S2. Change in anisotropy as a function of PhuS. *A.* apo-PhuS titration against the *prrF1-50* (No Fur), *prrF2-50* (Fur) and *prrF2-50* (AlgR) oligonucleotides as shown. *B.* holo-PhuS titration of *prrF1-50*. Experiments were performed in triplicate as described in the Experimental Procedures. The anisotropy, *r*, was plotted against protein concentration and the error is shown as the standard error of the mean (SEM).

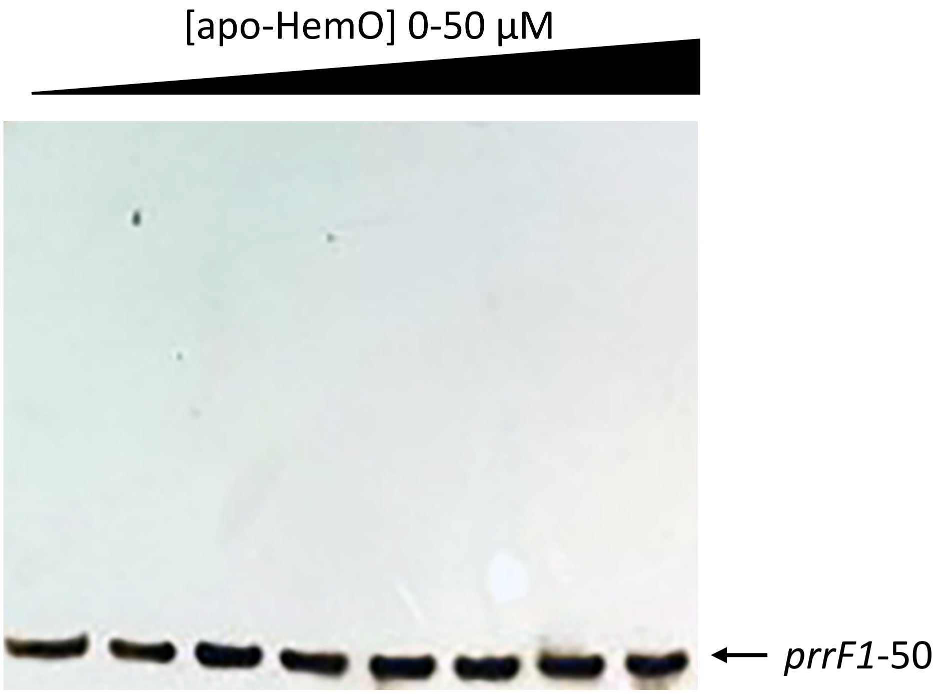

Figure S3. EMSA of apo- HemO binding to *prrF1*-50.. apo-HemO binding to 5'-biotin labeled *prrF1*-50. Experiments were performed as described in Experimental Procedures. All reactions contained a fixed concentration (30 pM) of labeled *prrF1*-50 and following incubation were run on 8% acrylamide gels and transferred to a nylon membrane and visualized by chemiluminescence.

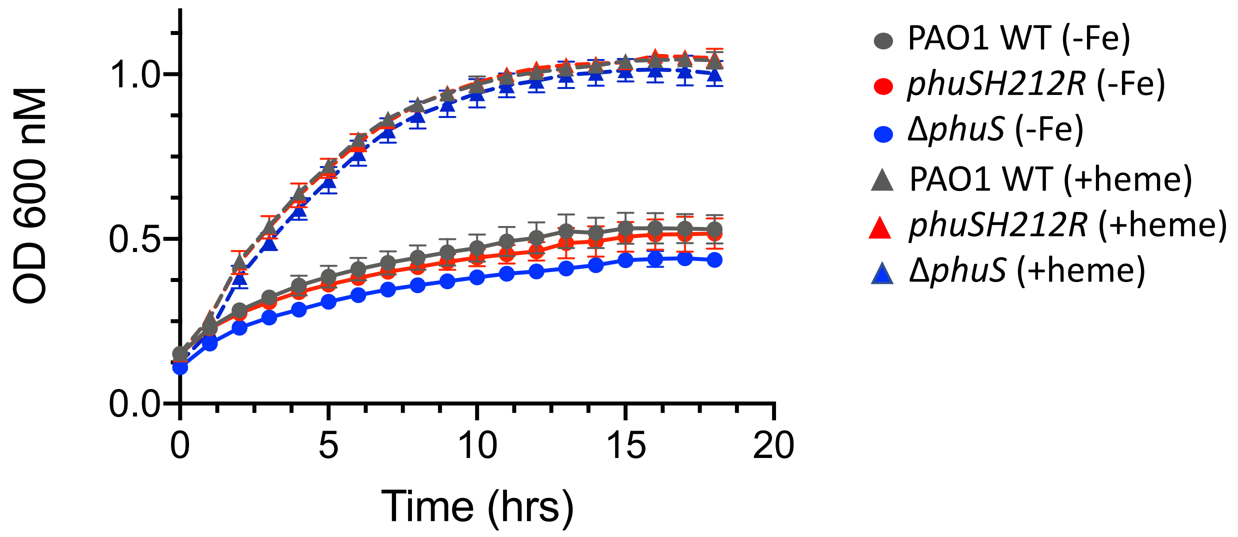

Figure S4. Growth curves for PAO1WT, *phuSH212R* and Δ*phuS* strains. Cells were grown at 37°C in iron-deplete media (M9) (circles) or M9 supplemented with 1 μM heme (triangles). The growth curves represent 3 biological replicates and the error bars represent the standard error of the mean (SEM). Strains as shown in the legend.

REFERENCES

1. Holloway, B. W. (1955) Genetic Recombination in *Pseudomonas aeruginosa*. *J. Gen. Microbiol.* **13**, 572-581
2. Barker, K. D., Barkovits, K., and Wilks, A. (2012) Metabolic flux of extracellular heme uptake in *Pseudomonas aeruginosa* is driven by the iron-regulated heme oxygenase (HemO). *J Biol Chem* **287**, 18342-18350
3. Lansky, I. B., Lukat-Rodgers, G. S., Block, D., Rodgers, K. R., Ratliff, M., and Wilks, A. (2006) The cytoplasmic heme-binding protein (PhuS) from the heme uptake system of *Pseudomonas aeruginosa* is an intracellular heme-trafficking protein to the delta-regioselective heme oxygenase. *J Biol Chem* **281**, 13652-13662
4. Deredge, D. J., Huang, W., Hui, C., Matsumura, H., Yue, Z., Moenne-Loccoz, P., Shen, J., Wintrode, P. L., and Wilks, A. (2017) Ligand-induced allostery in the interaction of the *Pseudomonas aeruginosa* heme binding protein with heme oxygenase. *Proc Natl Acad Sci U S A* **114**, 3421-3426
5. Pohl, E., Haller, J. C., Mijovilovich, A., Meyer-Klaucke, W., Garman, E., and Vasil, M. L. (2003) Architecture of a protein central to iron homeostasis: crystal structure and spectroscopic analysis of the ferric uptake regulator. *Mol Microbiol* **47**, 903-915
6. Ratliff, M., Zhu, W., Deshmukh, R., Wilks, A., and Stojiljkovic, I. (2001) Homologues of Neisserial Heme Oxygenase in Gram-Negative Bacteria: Degradation of Heme by the Product of thepigA Gene of *Pseudomonas aeruginosa*. *J Bacteriol* **183**, 6394-6403
7. Hoang, T. T., Karkhoff-Schweizer, R. R., Kutchma, A. J., and Schweizer, H. P. (1998) A broad-host-range Flp-FRT recombination system for site-specific excision of chromosomally-located DNA sequences: application for isolation of unmarked *Pseudomonas aeruginosa* mutants. *Gene* **212**, 77-86
